# Selection of housekeeping genes for use in quantitative reverse transcription PCR assays on the murine cornea

**Published:** 2010-06-11

**Authors:** Shengwei Ren, Feng Zhang, Changyou Li, Changkai Jia, Siyuan Li, Haijie Xi, Hongbo Zhang, Lingling Yang, Yiqiang Wang

**Affiliations:** Shandong Provincial Key Laboratory of Ophthalmology, Shandong Eye Institute, Qingdao, China

## Abstract

**Purpose:**

To evaluate the suitability of common housekeeping genes (HKGs) for use in quantitative reverse transcription PCR (qRT–PCR) assays of the cornea in various murine disease models.

**Methods:**

Corneal disease models studied were: 1) corneal neovascularization (CorNV) induced by suture or chemical burn, 2) corneal infection with *Candida albicans* or *Aspergillus fumigatus* by intrastromal injection of live spores, and 3) perforating corneal injury (PCI) in Balb/c mice or C57BL/6 mice. Expression of 8 HKGs (glyceraldehyde-3-phosphate dehydrogenase [*GAPDH*], beta-actin [*ACTB*], lactate dehydrogenase A [*LDHA*], ribosomal protein L5 [*RPL5*], ubiquitin C [*UBC*], peptidylprolyl isomerase A [*PPIA*], TATA-box binding protein [*TBP1*], and hypoxanthine guanine phosphoribosyl transferase [*HPRT1*]) in the cornea were measured at various time points by microarray hybridization or qRT–PCR and the data analyzed using geNorm and NormFinder.

**Results:**

Microarray results showed that under the CorNV condition the expression stability of the 8 HKGs decreased in order of *PPIA*>*RPL5*>*HPRT1*>*ACTB*>*UBC*>*TBP1*>*GAPDH*>*LDHA*. qRT–PCR analyses demonstrated that expression of none of the 8 HKGs remained stable under all conditions, while *GAPDH* and *ACTB* were among the least stably expressed markers under most conditions. Both geNorm and NormFinder analyses proposed best HKGs or HKG combinations that differ between the various models. NormFinder proposed *PPIA* as best HKG for three CorNV models and PCI model, as well as *UBC* for two fungal keratitis models. geNorm analysis demonstrated that a similar model in different mice strains or caused by different stimuli may require different HKGs or HKG pairs for the best normalization. Namely, geNorm proposed *PPIA* and *HRPT1* and *PPIA* and *RPL5* pairs for chemical burn-induced CorNV in Balb/c and C57BL/6 mice, respectively, while *UBC* and *HPRT1* and *UBC* and *LDHA* were best for *Candida* and *Aspergillus* induced keratitis in Balb/c mice, respectively.

**Conclusions:**

When qRT–PCR is designed for studies of gene expression in murine cornea, preselection of situation-specific reference genes is recommended. In the absence of knowledge about situation-specific HKGs, *PPIA* and *UBC*, either alone or in combination with *HPRT1* or *RPL5*, can be employed.

## Introduction

Quantitative PCR (q-PCR), also known as real time PCR, is being increasingly used in studies of diverse biologic processes due to its outstanding accuracy, broad dynamic range, high sensitivity, and high reproducibility. Unlike regular PCR protocols that require gel electrophoresis and imaging after the PCR reaction, q-PCR requires minimal post-PCR handling and thus is less time-consuming and less laborious. One of the main uses of PCR, when coupled with reverse transcription, is to measure gene expression at the mRNA level in various biologic samples. In such cases, either semi-quantitative reverse transcription PCR (RT–PCR) or the quantitative reverse transcription PCR (qRT–PCR) relies on normalization to a housekeeping gene (HKG) which is often referred to as the reference gene or internal control gene to ensure the accuracy of the assay. Ideally, expression of an HKG should show minimal variability between samples under different experimental conditions. However, it has been gradually realized that all HKGs, especially those traditional HKGs selected based on PCR experiments of earlier days, like beta-actin (*ACTB*) and glyceraldehyde-3-phosphate dehydrogenase (*GAPDH*), are not necessarily expressed at stable levels in all tissues/cells under all conditions [1-5]. Thus, the conclusions of many previous reports that were based on PCR results using these genes as HKGs might have to be reinterpreted to take into account the instability of HKGs expression. One study showed that the use of unsuitable reference genes resulted in 100 fold variance in apparent cytokine gene transcription [6]. Some studies propose the use of a combination of HKGs to minimize the potential risk implicit in the use of a single HKG [7], while others endeavor to identify novel “real” HKGs that are expressed at more stable levels in various tissues of multiple species [8-13]. Thus, the list of HKG candidates to choose from is growing rapidly. However, the suitability of such HKGs in studies of the cornea has not been re-addressed since the advent of qRT–PCR technology and the aforementioned improved knowledge of HKGs. In this paper, we establish models of experimental corneal neovascularization (CorNV), fungal keratitis (FK) and corneal wound in mice and study the expression patterns of three traditional HKGs (*ACTB*, lactate dehydrogenase [*LDHA*], and *GAPDH*), five new HKGs (ubiquitin C [*UBC*] [14], peptidylprolyl isomerase A [*PPIA*] [15], TATA-box binding protein [*TBP*] [16], hypoxanthine guanine phosphoribosyl transferase [*HPRT1*] [17], and ribosomal protein L5 [*RPL5*] [18]). Our results highlight the importance of choosing tissue-specific or condition-specific HKGs for each specific experimental system.

## Methods

### Animals

Female Balb/c and C57BL/6 mice (Chinese Academy of Medical Sciences, Beijing, China) were used at 6–8 weeks of age. All corneas were individually inspected under a slit lamp microscope before recruiting into experiments. The ARVO Statement for the Use of Animals in Ophthalmic and Vision Research was observed throughout the study, and only the right corneas were used for model induction and the left eyes were used as untreated controls.

### Experimental models for diseases of the cornea

All model protocols were performed under anesthesia with intraperitoneal chlorpromazine and ketamine plus topical application of Benoxil (Santen, Osaka, Japan). A total of three disease models in six groups of mice were used. For suture-induced CorNV (S-CorNV) induction, three interrupted stitches of 10–0 polypropylene suture (MANI Inc., Togichi, Japan) were placed in the corneas of Balb/c mice [19]. For chemical burn-induced CorNV (CB-CorNV) induction [20], a paper filter of 2 mm diameter soaked in 1.5 μl of 1 N NaOH was placed in the center of the corneas for 40 s, followed by rinsing with 10 ml of saline buffer. For fungal keratitis induction, 5×10^4^ live spores of *Candida albicans* or *Aspergillus fumigatus* in 0.5 μl were injected into the stroma of corneas as described previously [21]. The method of Oshima et al. [22] was used to make perforating corneal injury (PCI). In brief, a circular indentation was made with a 1.2 mm diameter trephine in the center of mouse corneas. Two perforating cuts, perpendicular to each other and reaching the circular mark at both ends, were made with a 20 guage paracentesis knife. Ofloxacin eye ointment was applied once immediately after the injury. With all models, the sacrificed eyes were checked daily under a slit lamp equipped with a digital camera.

### Isolation of total RNA

At each chosen time point, corneas were harvested for extraction of total RNA and the RNA was used for either microarray or qRT–PCR. The corneas were excised using a 2 mm diameter trephine and placed in ice-cold TRIzol reagent (Invitrogen, Gaithersburg, MD). Five model corneas from each group of mice were pooled and the untreated corneas from the same mice were used as control. Total RNA was extracted using isopropanol precipitation, and purified using NucleoSpin® RNA clean-up columns (MACHEREY-NAGEL, Düren, Germany). The quality and integrity of the RNA were confirmed by denaturing aldehyde agarose electrophoresis.

### Microarray analysis

Dual cRNA labeling with Cy5 and Cy3 fluorescence and microarray hybridizations were performed by Capital Bio Corporation using Capital Bio cRNA labeling kits and the Capital Bio 36 K Mouse Genome Oligo Array (Capital Bio, Beijing, China) [23]. In brief, the array comprises 35,852 70-mer oligonucleotide probes representing approximately 25,000 genes of Mouse Genome Version 4.0 (Operon Biotechnologies, Huntsville, AL). Cy5 and Cy3 were used to label cRNA of experimental and control groups, respectively. Two or three replicate arrays were used for each time point of each model. After hybridization, the arrays were scanned using a LuxScan 10KA (Capital Bio), and signals were processed with LuxScan 3.0 software (Capital Bio). Intra-array normalization was done using Lowess linearization method and inter-array normalization of the whole data set was performed according to the global means of Cy5 and Cy3 signals [24]. This microarray designates four HKGs for potential use, namely *ACTB*, *GAPDH*, *RPL5*, and *LDHA*. Each of these four HKGs is represented by 50 spots in the array. Normalized signal intensities were compared between experimental and control samples to evaluate the change in, or stability of, the expression level of each gene, including the HKGs.

### qRT–PCR analysis

For validation of HKGs using qRT–PCR, RNA from individual corneas in the same group were pooled to yield one RNA sample per group. One microgram total RNA from each pooled sample was reverse transcribed into cDNA using a PrimeScript RT Reagent Kit (TaKaRa Biotechnology [Dalian] Co., Ltd, Dalian, China) according to the protocol provided by the manufacturer. The expression levels of HKGs and one target gene (Table 1) were compared side by side using qRT–PCR with the TaqMan probes and primers (Table 2). Reactions for each sample were performed in triplicate using an ABI 7500 Detection System (Applied Biosystems, Foster City, CA) and a PCR protocol comprising an initial 10 min incubation at 95 °C followed by 40 cycles of 15 s at 95 °C and 1 min at 60 °C. The raw data were analyzed using SDS 7500 software (Applied Biosystems) and C_t_ values for each gene in each sample were determined for further analysis. The PCR efficiency of each primers/probe set was measured using Relative Expression Software Tool 2008 [25] and was found to be between 86% and 100.3%, reflecting the reliability of the qRT–PCR (Table 2).

**Table 1:** Summary of HKGs and target gene used in this study.

**Symbol**	**Gene name**	**Function**
Housekeeping genes		
*GAPDH*	Glyceraldehyde-3-phosphate dehydrogenase	Glycolytic enzyme
*ACTB*	Actin, beta	Cytoskeletal structural protein
*LDHA*	Lactate dehydrogenase A	Catalytic activity
*RPL5*	Ribosomal protein L5	Component of the 60S subunit of ribosome
*UBC*	Ubiquitin C	Possible involvement in protein catabolism
*PPIA*	Peptidylprolyl isomerase A	Catalyzes the cis-trans isomerization of proline imidic peptide bonds in oligopeptides and accelerates the folding of proteins
*TBP1*	TATA-box binding protein	General RNA polymerase II transcription factor
*HPRT1*	Hypoxanthine-guanine phosphoribosyl transferase	Purine synthesis in salvage pathway
Target gene		
*Tkt*	transketolase	calcium ion binding, corneal crystallin

**Table 2:** Characteristics of primers, probes, and PCR efficiencies of the housekeeping and target genes used in this study.

**Gene**	**Oligo**	**Sequence**	**Amplicon (bps)**	**PCR efficiency**
*GAPDH*NM_008084	F	TGTGTCCGTCGTGGATCTGA	77	91.3%
	R	CCTGCTTCACCACCTTCTTGA		
	P	CCGCCTGGAGAAACCTGCCAAGTATG		
*LDHA*NM_010699	F	ATCCCATTTCCACCATGATT	183	86%
	R	ACTGCAGCTCCTTCTGGATT		
	P	CAGGCGGGCCTCTTCCTCAG		
*ACTB*NM_007393	F	GCAAGCAGGAGTACGATGAG	148	92.7%
	R	CCATGCCAATGTTGTCTCTT		
	P	TCCATCGTGCACCGCAAGTG		
*UBC*NM_019639	F	ACCAGCAGAGGCTGATCTTT	110	88.7%
	R	ACCTCTGAGGCGAAGGACTA		
	P	CTGGAAGATGGCCGCACCCT		
*PPIA*NM_008907	F	AATGCTGGACCAAACACAAA	117	100.3%
	R	TTCCACAATGTTCATGCCTT		
	P	TGCTTGCCATCCAGCCATTCA		
*TBP1*NM_013684	F	ATCAACATCTCAGCAACCCA	187	97.1%
	R	TTGAAGCTGCGGTACAATTC		
	P	ACCACTGCACCGTTGCCAGG		
*HPRT1*NM_013556	F	GGCCAGACTTTGTTGGATTT	155	95.1%
	R	CAGATTCAACTTGCGCTCAT		
	P	TGACACAAACGTGATTCAAATCCCTG		
*RPL5*NM_016980	F	GGAAGCACATCATGGGTCAGA	70	91.8%
	R	TACGCATCTTCATCTTCCTCCATT		
	P	TGTGGCAGACTACATGCGCTACC		
*Tkt*NM_009388	F	GACAGTGCCCTTCTGCAGTACTT	65	101.1%
	R	CCATGCGAATCTGGTCGAA		
	P	CGCGGCCTTCTTCACACGGG		

F, forward primer; R, reverse primer; P, Probe, labeled as 5'-FAM, 3'-TAMRA.

### Analysis of HKG expression level stability

To evaluate the stability of expression of potential HKGs, non-normalized gene expression levels among the various experimental groups were analyzed using two programs, namely geNorm version 3.5 and NormFinder version 19, detailed principles and calculations of which are beyond this manuscript. Briefly, the geNorm works in Microsoft Excel and determines the most stable reference genes from a set of tested genes in a given cDNA sample panel. geNorm calculates the gene expression stability measure M for a reference gene as the average pairwise variation V for that gene with all other tested reference genes. Stepwise exclusion of the gene with the highest M value allows ranking of the tested genes according to their expression stability [26]. NormFinder uses a solid statistical framework to estimate not only the overall expression variation of the candidate normalization genes, but also the variation between sample subgroups of the sample set (e.g., control and model samples). NormFinder provides a stability value for each gene, which is a direct measure for the estimated expression variation enabling the user to evaluate the systematic error introduced when using the gene for normalization. It ranks the set of candidate genes according to their expression stability in a given sample set and given experimental design [27]. Though using different computing modes and formulae, both programs propose a single best HKG or pair of HKGs. The instructions provided with each software were followed when inputting the qRT–PCR data, fetching the output, and interpreting the analysis results.

### Test of suitability of selected HKGs in corneal disease models using target gene

To test the suitability of HKGs proposed by geNorm or NormFinder and to demonstrate the importance of choosing a suitable HKG or HKG pair when determining the level of target gene expression in different corneal disease models, expression of a target gene, namely transketolase (*Tkt*), and 8 HKGs in the same samples was monitored by qRT–PCR. *Tkt* encodes a dominant enzyme of the cornea [28] whose expression is known to change upon exogenous stimulus [29]. The apparent relative expression level of *Tkt* in experimental corneas compared to that in control corneas was calculated from either from 1/(2^ΔCt^) when no normalization was applied, or from 1/(2^ΔΔCt^) when normalization against various HKGs was applied, where ΔC_t_=C_tTkt•model_-C_tTkt•control_ and ΔΔC_t_=(C_tTkt•model_ -C_tHKG•model_)-(C_tTkt•control_ -C_tHKG•control_).

## Results and Discussion

### Establishment of CorNV, FK, and injury models

**Figure 1:**
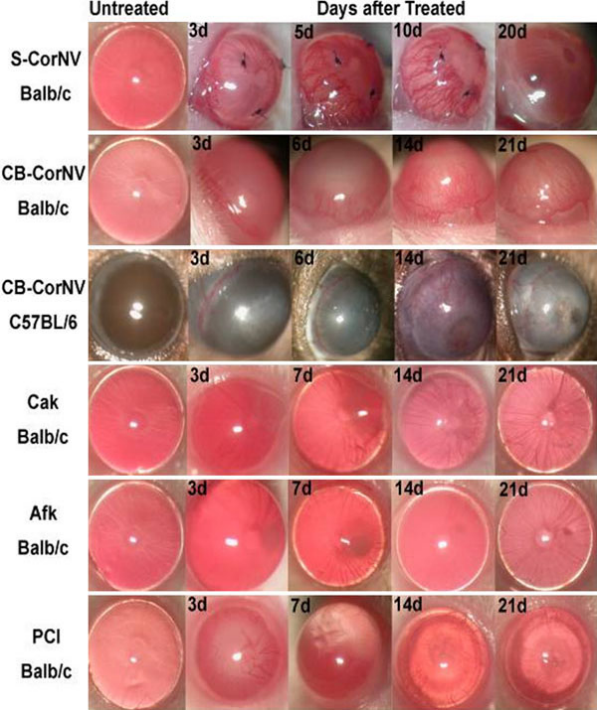
Macroscopic manifestation of various corneal disease models. Please pay close attention to the similarity or difference between related models, like corneal neovascularization induced in same animal strain by different method (S-CorNV and CB-CorNV in Balb/c mice) or induced in different strain with same method (CB-CorNV in Balb/c and C57BL/6 mice). Infection with different pathogen strains caused similar disease but with different severity (CaK and AfK in Balb/c mice).

Inflammatory CorNV can be effectively induced by both suture and chemical burn, however, its development in these two models are different. For example, S-CorNV and CB-CorNV vessels in Balb/c mice reached maximum length around day 10 (D10) and D14, respectively. After removing the suture at D10, the vessels retract rapidly and the cornea returned to transparency by around D20. As with fungal keratitis, both *Candida albicans* keratitis (CaK) and *Aspergillus fumigatu*s keratitis (AfK) caused the most severe disease around D10, and the damage started to recover thereafter. For perforating injury to the cornea, the anterior chamber started to reconstitute at D7 through self-healing but a scar was still apparent in the injured area even on D21. The general appearance of the corneas under slit lamp at the analyzed times is summarized in Figure 1.

### Expression levels of HKGs in CorNV models as assessed by microarray hybridization

**Figure 2:**
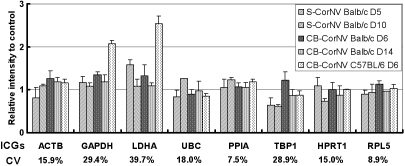
Changes in expression of the 8 HKGs in murine corneas with experimental CorNV as assessed by microarray. The ratios were obtained by comparing the normalized fluorescence intensity of experimental corneas to that of the controls. In this commercial microarray, *ACTB*, *LDHA*, *GAPDH*, and *RPL5* are used as HKGs, thus each is represented by 50 spots in the array. The average of these 50 signals was used to calculate the average and standard error for each group and this was used for comparison with the other four genes (viz. *UBC*, *PPIA*, *TBP1*, and *HPRT1*, which are represented by only one spot in the array). The data presented (mean±standard deviation) were obtained from three (for S-CorNV Balb/c D5 and CB-CorNV Balb/c D6 groups) or two (for the other three groups) arrays. The coefficient of variation (CV) was obtained by dividing the standard deviation by the mean in each model. S-CorNV Balb/c D5: suture-induced CorNV in Balb/c mice, day 5; S-CorNV Balb/c D10: suture-induced CorNV in Balb/c mice, day 10; CB-CorNV Balb/c D6: chemical burn-induced CorNV in Balb/c mice, day 6; CB-CorNV Balb/c D14: chemical burn-induced CorNV in Balb/c mice, day 14; CB-CorNV C57Bl/6 D6: chemical burn-induced CorNV in C57Bl/6 mice, day 6.

In addition to high throughput gene expression profiling, microarray technology has also been used to identify novel HKGs that could be employed as normalization controls in qRT–PCR [30,31]. We assayed the expression of the 8 HKGs in 12 microarrays originally used for differential gene expression profiling in the CorNV models. Statistical ANOVA showed that *PPIA* and *RPL5* were among the genes with most stable expression levels (i.e., lowest CV) while *LDHA* and *GAPDH* were the genes with the most variable expression in the CorNV model (Figure 2). In fact, *ACTB*, *GAPDH*, *RPL5*, and *LDHA* are designated reference genes for potential use in this microarray system. Our findings here demonstrated that great caution should also be taken when use HKGs for normalization during microarray assays.

### Expression levels of HKGs as assessed by qRT–PCR

**Figure 3:**
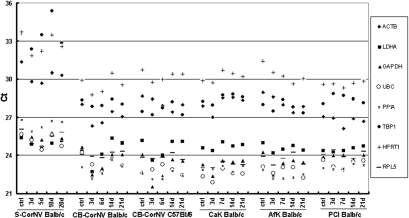
Raw C_t_ data for 8 candidate HKGs in each corneal disease model obtained using qRT–PCR. It should be noted that each point represents the mean of reactions performed in triplicate for each cDNA sample and for each gene.

We next examined the relative expression levels of the HKGs in all corneal disease models by qRT–PCR. Direct comparison of C_t_ values clearly showed that all 8 HKGS displayed significant changes in expression level under at least one condition (Figure 3). For instance, with the exception of the PCI model, *GAPDH* expression levels changed in all five models compared with the controls. In the CB-CorNV C57Bl/6 model, *GAPDH* expression at day 3 was about 5.8 fold that of the control. Expression of *ACTB* also changed in all models, though to a lesser extent than *GAPDH*. These results confirmed the findings by others using other tissues that *ACTB* and *GAPDH* were not suitable for normalizing gene expression [3,5,32], and demonstrated the necessity for determining the most suitable HKG for each corneal disease model.

### Selection of optimal HKGs

**Figure 4:**
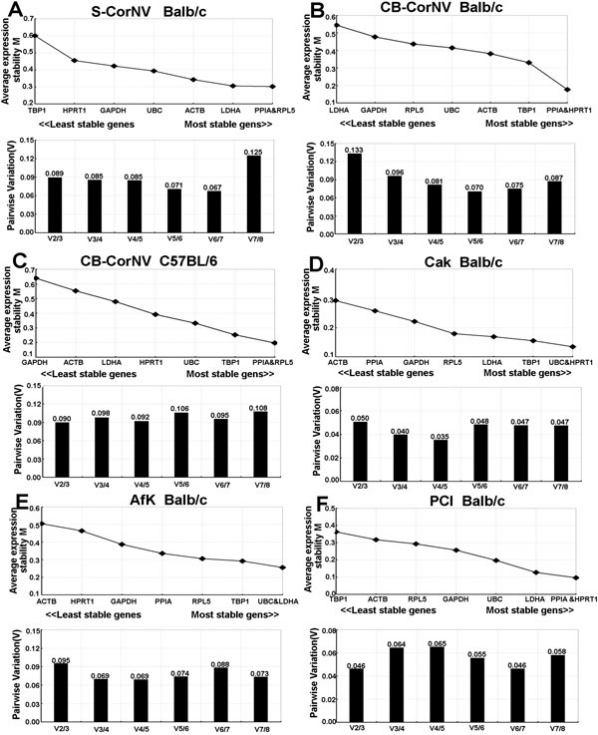
Average expression level stability (M) and pairwise variation (V) of 8 HKGs as assessed by geNorm analysis. The genes with the lower M values are considered to have more stable expression levels. Pairwise variation was used to determine the optimal number of HKGs required for normalization. According to the algorithm and instructions provided with the software, a cutoff of 0.15 for V was used. It was apparent from analysis of all studied models that a combination of two HKGs is sufficient for normalization.

Software programs geNorm and NormFinder rely on different computational algorithms (the details of which are outside the scope of this paper) and are often used in combination for selection of HKGs in various studies. However, these programs often identify different HKGs within the same batch of data [27,33]. geNorm analysis of the above qRT–PCR data demonstrated that a combination of two HKGs would be sufficient for normalization, and an optimal pair was proposed for each model (Figure 4). Analysis of the same data by NormFinder revealed a single optimal HKG for each condition (Table 3).

**Table 3:** Stability of the expression levels of various HKGs as revealed by NormFinder.

**Gene name**	**S-CorNV Balb/c**	**CB-CorNV Balb/c**	**CB-CorNV C57BL/6**	**CaK Balb/c**	**AfK Balb/c**	**PCI Balb/c**
*ACTB*	0.229	0.184	0.477	0.257	0.394	0.225
*LDHA*	0.243	0.473	0.337	0.066	0.117	0.117
*GAPDH*	0.276	0.309	0.587	0.173	0.337	0.150
*UBC*	0.239	0.324	0.407	0.021	0.089	0.208
*PPIA*	0.107	0.088	0.068	0.256	0.129	0.096
*TBP1*	0.686	0.231	0.274	0.064	0.232	0.314
*HPRT1*	0.237	0.126	0.095	0.084	0.352	0.100
*RPL5*	0.240	0.326	0.160	0.136	0.296	0.194
Best gene	*PPIA*	*PPIA*	*PPIA*	*UBC*	*UBC*	*PPIA*

As summarized in Table 4, of the six optimal pairs of HKGs suggested by the geNorm program, *PPIA* and *HPRT1* were chosen 4 and 3 times, respectively, while *UBC* and *RPL5* were chosen 2 times each. Similarly, NormFinder analysis of the 8 proposed HKGs showed that *PPIA* and *UBC* were the optimal HKGs when using a single HKG. Neither *ACTB* nor *GAPDH* was chosen as optimal HKG in any of the models. Since all three CorNV models and the PCI model used here involve physical or chemical damage to the cornea, it is unlikely to be a coincidence that analyses of all CorNV models and the PCI model all indicate *PPIA* as the optimal HKG. Similarly, analyses of the two keratitis models induced by different fungi both indicate the same optimal HKG (viz. *UBC*).

**Table 4:** Summary of the optimal candidate HKG(s) for each model.

**Disease model**	NormFinder	geNorm
S-CorNV Balb/c	*PPIA*	*PPIA* & *RPL5*
CB-CorNV Balb/c	*PPIA*	*PPIA* & *HPRT1*
CB-CorNV C57BL/6	*PPIA*	*PPIA* & *RPL5*
CaK Balb/c	*UBC*	*UBC* & *HPRT1*
AfK Balb/c	*UBC*	*UBC* & *LDHA*
PCI Balb/c	*PPIA*	*PPIA* & *HPRT1*

### The influence that choice of HKG has on the apparent expression level of target genes

**Figure 5:**
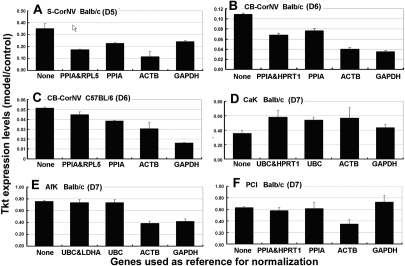
An example of the influence that choice of HKGs can have on the apparent changes in *Tkt* gene expression levels. Only data from the second time point of each treatment is shown, but is representative of that disease model, i.e., D5 for S-CorNV, D6 for CB-CorNV Balb/c and CB-CorNV C57Bl/6, D7 for CaK, AfK and PCI. The y-axis is for ratio of the *Tkt* expression levels in disease model corneas to that in control corneas that were obtained via direct comparison of non-normalized C_t_ (*none*) or C_t_ normalized using each optimal HKG or HKG pair proposed by geNorm or NormFinder, plus *ACTB* and *GAPDH*. Please note that the optimal HKGs or HKG pairs were different among models as shown in Table 4. When a HKG pair was used, the geometric means of relative expression levels against each HKG was used.

Lastly, we demonstrated the importance of choosing a suitable HKG or HKG pair when determining the level of target gene expression by qRT–PCR in different corneal disease models by comparing the apparent expression changes in various models when different HKGs were used. Although the actual changes could not be easily determined, it can be seen from Figure 5 that normalization of the qRT–PCR data against the optimal HKG suggested by either NormFinder or geNorm, gave comparable relative expression ratios of *Tkt* in most models. On the contrary, normalization against either *ACTB* or *GAPDH* resulted in relative expression ratios that were significantly different from each other, implying that *ACTB* and *GAPDH* are not suitable HKGs for these studies. We hope this finding will alert workers in the ophthalmology and visual science fields of the dangers of underestimating the importance of validating potential HKGs for qRT–PCR. Among the total of 37 papers published in IOVS or Molecular Vision between the years 2000 and 2009 that address CorNV or FK using RT–PCR, 22 papers used *GAPDH*, 14 papers used *ACTB*, and 1 paper used 18s rRNA as HKG for normalization (detailed list of papers not shown). In fact, most PCR studies reported in the ophthalmology journals are still using these two traditional HKGs without pre-selection.

To the best of our knowledge, this is the first report to show that the optimal HKGs or HKG pairs for a study may vary according to the specific details of the samples studied. In other words, the optimal HKG or HKG pair is condition-specific. The “condition” here may refer to the specific status of the cells, tissues, organs, animal strains, or the physiochemical properties of a stimulus, the concentration and persistence of a factor introduced into the system, and so on. For example, S-CorNV and CB-CorNV models in the same strain of mice (Balb/c mice) require different HKG pairs, as do the same CorNV inducer (chemical burn) in different mice strains (Balb/c versus C57Bl/6). Similarly, keratitis caused by infection with different fungi also requires different HKG pairs (Figure 3, Table 4). Thus, although *PPIA*, *UBC*, *HPRT1*, and *RPL5* appear to be the optimal choice of HKGs for the gene expression studies described here, one cannot assume they would be suitable HKGs or HKG pair for any other models (like bacterial keratitis, dry eye, etc) that differ in any way from the models used in this study.

In summary, our data suggests that it is desirable to determine the suitability of any common HKGs or to specifically search for a suitable HKG whenever a new model is to be used for the murine cornea. To generalize, pre-selection of HKGs should be a routine step for any new experimental system in a laboratory. In case there is a lack of information about possible HKGs that are available and a conventional HKG or HKG pair has to be used without pre-selection, *PPIA* or *UBC*, either alone or in combination with *HPRT1* or *RPL5*, are recommended. Again, due to numerous differences in gene expression between human and murine species, the applicability of the above conclusions needs to be verified if human corneas are to be studied with qRT–PCR methods.
